# The association between psychosocial stress, interpersonal sensitivity, social withdrawal and psychosis relapse: a systematic review

**DOI:** 10.1038/s41537-023-00349-w

**Published:** 2023-04-10

**Authors:** A. Almuqrin, A. Georgiades, K. Mouhitzadeh, P. Rubinic, A. Mechelli, S. Tognin

**Affiliations:** 1 Department of Psychosis Studies, Institute of Psychiatry, Psychology, and Neuroscience (IoPPN), King’s College London, London, UK; 2 Department of Health Sciences, College of Health and Rehabilitation Sciences, Princess Nourah bint Abdulrahman University, Riyadh, Saudi Arabia; 3 Brent Early Intervention Service, 27-29 Fairlight Avenue, London, NW10 8AL, CNWL, NHS Foundation Trust, London, UK

**Keywords:** Psychosis, Human behaviour

## Abstract

Psychosis is associated with a high risk of relapse, with 67% of clients relapsing within one year following a first episode. In light of the high personal, social, and healthcare costs of the illness, it is paramount to understand the risk factors associated with psychosis relapse. The current systematic review aims to critically review the role of psychosocial stress in psychosis relapse in individuals with an established psychotic disorder. This review systematically searched Ovid (PsycINFO, EMBASE, MEDLINE) literature databases from inception until 28th February 2022. Sixteen studies were eligible for inclusion. Most studies found that individuals with psychosis demonstrate high levels of psychosocial stress and are more likely to be socially withdrawn compared to healthy controls or other clinical presentations. Most studies reported a statistically significant association between psychosocial stress and psychosis relapse, as well as between social withdrawal and psychosis relapse. However, no studies examined the association between high levels of interpersonal sensitivity and psychosis relapse. Individuals with psychosis tend to experience high levels of psychosocial stress and social withdrawal, and these appear to increase the risk of psychosis relapse. Due to high levels of heterogeneity within the literature, we could only conduct a narrative synthesis of the findings. Future studies would benefit from employing a meta-analytic approach.

## Introduction

Psychosis is a reality distortion experience characterized by delusions, hallucinations, and disorganized thinking^1^. It is associated with a high risk of relapse, with ~67% of clients relapsing within one year following a first episode^2^. In addition to causing distress to patients and their families, psychosis relapse has been posited to exert adverse effects on underlying neurobiological processes, which in turn can negatively affect the long-term course of the illness^3^. Relapse has also been associated with increased risk of harm to self and others, which causes disruption to ones occupational, educational, and relationship roles^4^. Relapse can also increase perceptions of stigma, leading to increased isolation^3^. In addition to the distress and disability caused by psychosis, there is an economic burden associated with the illness as treating individuals with psychosis after a symptom relapse costs four times more than treating individuals with a stable course of the disorder^1^. These neurobiological, psychosocial, and healthcare consequences highlight the importance of understanding the risk factors associated with psychosis relapse.

Gene-environment interactions have been found to play a significant role in psychosis relapse^5,6^. Indeed, there is considerable evidence that a combination of genetic and environmental/psychosocial factors lead to the dysregulation of the mesolimbic dopamine pathway and of the hypothalamic-pituitary-adrenal axis. This subsequently results in an elevated sensitivity to psychosocial stressors and an increased risk of psychosis relapse^5–9^. While several studies have examined the impact of genetic risk factors^8,10^, research examining the impact of psychosocial risk factors on psychosis relapse is limited^5^.

*Psychosocial stress* is a potential social risk factor for psychosis relapse that is gaining interest. It has been defined as an emotional, physical, and psychological reaction to social stressors^11^. Social stressors can be grouped into three categories: chronic stressors (e.g., long-term unemployment), life events (e.g., homelessness), and minor daily hassles (e.g., offensive comments)^12^. Social stressors have been frequently reported prior to an episode of psychosis^13,14^, and a high number of stressful events tend to precede the onset of frank psychosis^15,16^. These findings are consistent with the stress-vulnerability model, which states that cumulative exposure to psychosocial stressors interacts with ones bio-psychosocial vulnerability to gives rise to mental health difficulties^17^. Furthermore, individuals with psychosis have been found to report a high level of psychosocial stress compared to healthy individuals^18–20^. These findings indicate that social stressors are common in individuals with psychosis.

The role of psychosocial stress in psychosis relapse has been suggested by previous studies^21,22^. Indeed, an association between high levels of psychosocial stress and psychosis relapse has been consistently reported^23–25^. This finding is also in line with recent retrospective cross-sectional studies reporting that relapse appears to be related to psychosocial stressors, such as moving home and ethnic minority status^21,22^. Nevertheless, this association’s direction is unclear, as many studies have found that individuals with psychosis tend to show heightened sensitivity to stress^6,26^. In other words, patients with psychosis may present with heightened stress sensitivity and/or might be exposed to a higher number of stressful events compared to the healthy population. These are distinct yet related aspects which can be individual targets of psychological interventions^27,28^. In addition, a few earlier studies have reported negative findings, suggesting that there is no association between psychosocial stress and psychosis relapse^29,30^.

Some of the above inconsistencies might be explained by individual characteristics such as *interpersonal sensitivity*—a personality trait, referring to the excessive awareness of the emotions and behaviour of others^31^, which has been implicated in the onset and maintenance of psychosis^32,33^. It has also been found that interpersonal sensitivity is prominent in the prodromal stages of the illness, among individuals at clinical high risk of psychosis (CHR)^34,35^ and that this trait was more prominent in CHR individuals compared to healthy controls^31,36^. Taken together, these studies suggest that individuals at risk of, and with established psychosis report a high level of interpersonal sensitivity, and that this may be associated with onset of psychosis and psychosis relapse. However, the association between interpersonal sensitivity and psychosis relapse has not been fully explored yet.

Alongside personality traits, social behaviours such as *social withdrawal*, have been studied in association with psychosis relapse. Social withdrawal refers to one’s withdrawal from interpersonal/social relationships accompanied by detachment and an apathetic attitude^37^. Social withdrawal is one of the negative symptoms of psychosis that, at least in part, explains the small social network observed in individuals suffering from psychosis^38^. Social withdrawal also appears to be positively linked with psychosis relapse^39,40^. This finding is in line with the bio ecosystem theory of negative symptoms that explains the impact of several environmental factors (i.e., socio-cultural factors) on negative symptoms (i.e., social withdrawal) and psychosis relapse^41^. It is still unclear whether social withdrawal progressively precedes psychosis relapse (and can therefore be potentially used as a predictor of relapse), is a negative symptom frequently observed in individuals who are experiencing an episode of psychosis, or a consequence of the illness due to self-stigma or avoidant coping. Social withdrawal, in all its manifestations, could therefore be an intervention target after an FEP to break vicious cycles of avoidance and potentially prevent reoccurrence of symptoms.

Several systematic reviews have examined the association between psychosocial stress and psychosis relapse in individuals with psychosis, however these reviews have several limitations. For example, as some were conducted before the millennium^23,25^ they did not include more reliable and accurate measurement tools (e.g., ecological momentary assessment) that could improve the ecological validity of findings. Other reviews have been found to be inconclusive^42^, or only included observational studies^43^. This did not allow to clarify the relationship between psychosocial stress and psychosis relapse. Previous reviews have also used different definitions of relapse. For example, Petros and colleagues have defined relapse as hospitalization^44^ and have excluded alternative definitions (e.g., exacerbation/return of symptoms without hospitalization). Other reviews have examined psychosocial stress focusing on a specific stressor, (e.g., childhood trauma)^44^, which limits the generalizability of the findings as the term psychosocial stress might refer to a wide range of different stressors (e.g., significant life events and minor daily hassles). It is also important to note that childhood trauma cannot be equated or treated similarly to arguments, moving home, or the experience of uncontrollable daily hassles.

Finally, only one review has examined psychosocial stress focusing on different stressors^45^. However, it included studies that induced psychosocial stress experimentally, which limits the findings’ generalizability as it excluded naturally occurring stressors within one’s daily environment.

**Fig. 1:**
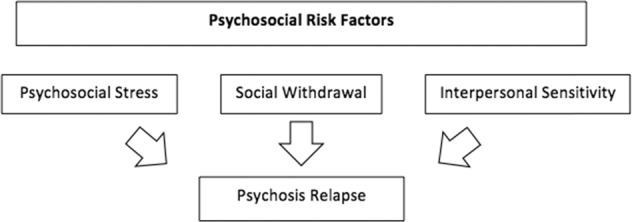
FEP Relapse Model: A causal model linking psychosocial stress, interpersonal sensitivity, and social withdrawal with the risk of psychosis relapse. We propose the following *Psychosis Relapse Model*. This model suggests that a combination of psychosocial stress, social withdrawal, and interpersonal sensitivity increase risk for psychosis relapse and could be targets of preventive strategies.

The current systematic review aims to overcome some of the above limitations by critically considering different social risk factors, specifically, psychosocial stress, interpersonal sensitivity, and social withdrawal, and their associations with psychosis relapse in individuals with established psychotic disorder (Fig. 1). A better understanding of psychosis’ psychosocial risk factors might allow mental health professionals to intervene early and to apply available preventative strategies before the re-emergence of symptoms.

## Results

### Search results

**Fig. 2:**
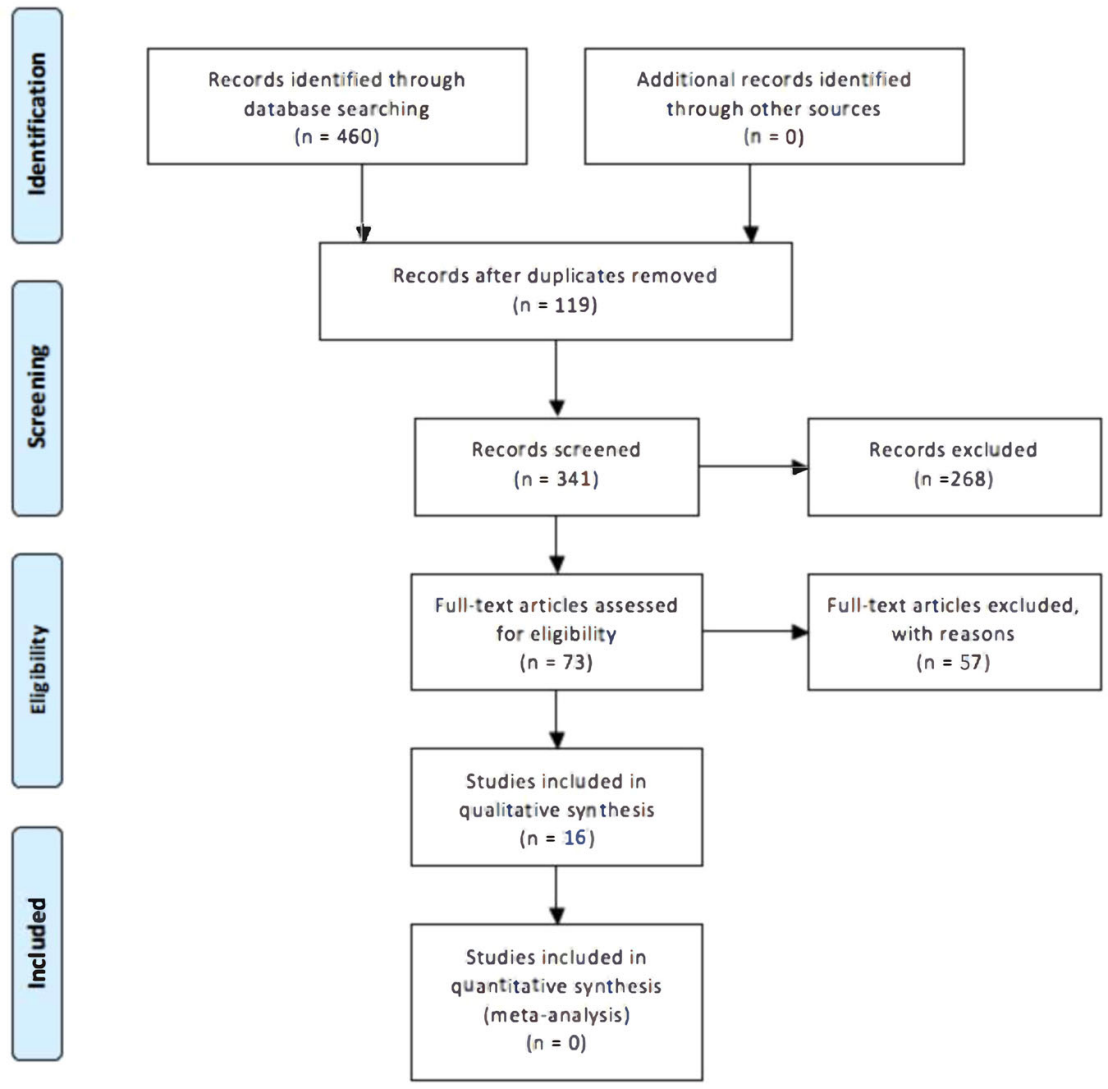
PRISMA Flow Diagram.

The PRISMA flow diagram of the search results is presented in Fig. 2, where 460 eligible studies were identified for the initial screening. Of these, 119 were duplicates and thus removed, leaving 341 studies for the initial screening. Out of these, 268 were excluded due to being irrelevant to the research questions, leaving 73 studies for full-text retrieval. During the full-text retrieval, 15 studies were excluded due to being qualitative, reviews, validity studies, or being inaccessible in English. The remaining 58 studies were thoroughly reviewed, and of those, 40 were excluded due to not meeting the inclusion criteria. Further, two additional studies were excluded due to insufficient data. A final number of 16 studies matched the inclusion criteria for this review.

### Study characteristics

The characteristics of the 16 included studies are presented in Table 1. These studies were published between 1971 and 2021 and conducted in eight countries. These studies adopted different research designs: 11 were cohort studies, 5 were cross-sectional studies.

**Table 1:** Characteristics of included studies.

Author (date) location	Study design and total sample size (gender; male and female)	Patient group age in years (mean age ± SD)		Diagnosis and manual used	Ethnicity (%)	Socio economic status	Measure of clinical outcome (relapse)	Measures	Main findings and statistical analysis conducted
Buck et al. (2019) USA	Randomised controlled trial *N* = 61 (M: 36, F: 25)	37.11 (±13.85)		26 schizophrenia, 26 schizoaffective, 9 psychosis NOS	17 Hispanic/Latino, 44 Non-Hispanic/non-Latino	72.13% unemployed	8-point criteria, including hospitalisation (Csernansky et al. 2002)		Exploratory data analytic framework For 20 participants, there were 27 relapse events (majority were hospitalisations, *n* = 22). Relapse was significantly associated with the total number of incoming and outgoing messages as well as with the number and duration of outgoing phone calls.
Camacho et al. (2012) USA	Cross-sectional *N* = 451 M: 51%		Although patients’ charts were reviewed to extract this information, it was not reported in the full text DSM-IV		265 (58) Hispanics, 36 (8) African Americans, 133 (29) Caucasian, 6 (1.3) Asians, missing data for 12 cases (2.6)		Emergency hospitalisation	Patients’ charts reviewed for stressors	Chi-square and multivariate logistic regression analysis Those with a psychotic disorder had significantly higher rates of hospitalisation (*P* < 0.01) From the total 451, 77 (17%) of patients were hospitalised. There were ethnic differences in the rates of hospitalisation with African Americans having the highest *P* < 0.01) and Hispanics the lowest *P* = 0.04) The rate of hospitalisation increased in patients who were homeless (*P* < 0.05) The rates of hospitalisation were lower in the presence of conflict with one’s partner/family (*P* < 0.01).
Caton & Goldstein (1984) USA	Longitudinal cohort (1 year) *N* = 119 M and F equal representation	34	119 Schizophrenia		60.5% American Black, 20% Hispanic	Social classes IV and V	Hospitalisation	Interview CCS (Caton et al. 1981)	ANOVA Change of address was associated with high interpersonal stress Level of social isolation was associated with change of address High average number of address changes was associated with greater number of relapses
Cechnicki & Wojciechowska (2008) Poland	Longitudinal cohort *N* = 64 (M: 28, F: 36)	32	64 Schizophrenia DSM-III			38 employed, 11 students, 4 on sickness/disability benefits and part-time work, 3 on benefits did not work, 8 unemployed without benefits	Hospitalisation/intensity of symptoms	Bizon’s questionnaire (2001; support network)	Spearman’s correlation coefficient Patients with larger social support networks experience shorter hospitalisations (*p* = −0.21) and have fewer relapses (*p* < 0.05) as well as less severe illness prognosis (*p* < 0.05). Fewer outpatient care hospitalisations were observed in those with large social support networks (*p* < 0.01). Larger support networks were also correlated with better employment rates (*p* < 0.05) and family functioning (less burden (*p* = 0.32), better adaptation to illness (*p* = 0.14), better family attitudes towards patients as less criticism/rejection experienced by patients; *p* = 0.23).
Chakraborty et al. (2011) UK	Longitudinal cohort (1-year follow-up) *N* = 110 (M:65; F:45)	43 (±14.5)	80 schizophrenia, 30 schizoaffective DSM		15 UK 83 Caribbean 7 African 5 others	97.3% unemployed	Hospitalisation (number of days, number of admissions, length of stay)	PRS (McNeilly et al. 1996)	Regression analysis Perceiving racism over the course of one-year positively predicted the days spent in hospital (*p* = 0.010). Perceiving racism over the course of one-year also predicted a longer duration of hospital stay.
Chu et al. (1985) USA	Cohort *N* = 128 (M: 69, F: 59)	22.56	128 Schizophrenia DSM-II		128 Black		Hospitalisation	Interview with patients and families	Multivariate analysis, stepwise regression, chi-squared If patients were not told that they would improve after discharge, they were more likely to be rehospitalised (*p* < 0.025). Patients with better social support during initial hospitalisation were less likely to be rehospitalised (*p* < 0.05). Patients who engaged in community activities, before (*p* < 0.05) and during (*p* < 0.01) hospitalisation were less likely to be rehospitalised. Patients whose family members had more helpful attitudes and behaviours towards their illness were less likely to be rehospitalised (*p* < 0.05).
Docherty et al. (2011) UK	Longitudinal *N* = 27 (M: 17, F: 10)	35 (±8.9) 18–55	27 schizophrenia DSM-IV		21 Caucasian, 6 African Americans		Symptom exacerbation	PCS (Hooley & Teasdale, 1989) CFI (Kazarian et al. 1990)	MANOVA and 3-way mixed-design ANOVA Patients with higher or lower levels of criticism from most influential others (MIO) did not significantly differ in terms of total symptoms at baseline. Patients with high critical MIO had higher levels of psychotic symptoms over time and those with low levels of critical MIO exhibited decreased psychotic symptoms (*p* = 0.03) large effect size) Being anxious and having high critical MIO resulted in the higher levels of symptom exacerbation (medium/large effect size)
Hazelgrove et al. (2021) UK	Prospective longitudinal *N* = 112 (F: 112)	32.7 (±5.5)	51 at risk of postpartum psychosis (PPP) (6 schizoaffective, 4 previous PPP episode) DSM-IV 28% on antipsychotics		31% White		Hospitalisation or exacerbation of symptoms	CECA (Bifulco et al. 2005) LTE-Q (Brugha & Cragg, 1990) CAS - Pregnancy Version (Hegarty, 2007)	Independent samples *t*-test, Mann–Whitney *U* test, Pearson’s chi-square test Of those at risk of an episode, 22 (43.1%) relapsed and were more likely that those who did not relapse to have endured severe childhood maltreatment (*p* = 0.04), and to have heightened sensitivity to stressful life events (*p* = 0.04) but did not experience more stressful life events during their pregnancy Those who relapsed were more likely to be unemployed (*p* = 0.01) Compared to HCs, those at risk experienced more severe childhood maltreatment (*p* < 0.001), including parental loss/separation (*p* = 0.03) They were also more likely to have experienced stressful events during pregnancy (*p* < 0.001)
Hultman et al. (1997) Sweden	Longitudinal cohort (9 months) *N* = 42 (M: 33, F: 9)	32.3 (±8.3)	42 Schizophrenia 40 on antipsychotics				Hospitalisation due to exacerbation of symptoms	LEDS (Brown & Harris, 1978) ISSI (Henderson et al. 1980)	T-tests, chi-squared, Spearman rank correlation, Wilcoxon rank sum test Low social support levels were observed. Patients coped with stress by socially withdrawing but those with high support levels tended to not use withdrawal strategies 36% of the total sample relapsed and 40% of the life-event screened group relapsed at 9-month follow-up. 50% of those who relapsed experienced one independent stressful life event in the three weeks prior to their hospitalisation (including property damage, death/illness in family, changes at work or redundancies) (*p* < 0.05) Socially isolated patients who were satisfied with their social contacts were more likely to relapse than those who were socially isolated but wanted to increase their social contact (*p* < 0.05). Having someone to support them buffered against the impact of the stressful life events (63% with low social support relapsed and 10% with high social support relapsed) and Fisher’s exact test showed significance (*p* < 0.05). Being able to cope by talking to someone significantly lowered risk of relapse given the life-event occurring (*p* < 0.001).
Krupinski et al. (1971) Australia	Cohort *N* = 127 (M:56, F: 71)		127 Schizophrenia ICD		31 Southern Europe		Hospitalisation	Interview with patients and families	Patients born in southern Europe (38.7%) experienced significant deterioration than others (18.1%; *p* < 0.05). In 22 acutely psychosis patients, only 5 (22.7%) showed improvement whereas those with chronic schizophrenia showed more improvements (37.1%). Only 26 (20.5%) reported employment. Of the 77 patients with deteriorated conditions, 22 (73.3%) reported little to no social life (*p* < 0.01). There was a significant association between relationships and relapse (*p* < 0.05) Family attitudes was associated with relapse: accepting patients with warmth resulted in 56.7% improving Of 30 patients with financial problems, 53.3% relapsed compared to those without (14.4%; *p* < 0.0001)
Lahniers & White (1976) USA	Cross-sectional *N* = 116	M: 32.2 F: 37.6	47 Schizophrenia DSM-II				Hospitalisation	SSRQ (Brown & Birley, 1970)	Univariate ANOVA No significant findings were found to suggest that the stressful life events resulted in relapse (hospitalisation)
Malla et al. (1990) Canada	Longitudinal cohort (1-year follow-up) *N* = 21 (57% M)	M: 26.6 F: 40.2	21 Schizophrenia DSM-II On antipsychotics				Symptom exacerbation	PERI (major life events; Dohrenwend et al., 1978) Minor daily events/changes also reported	Two-tailed *t*-tests 33% relapsed during the follow-up, and had a higher total number of events compared to those who did not relapse A significant difference between the average major and minor life events was found between those who relapsed and those who did not (*M* = 6.2 vs 3.5, *P* < 0.05). Those who relapsed had a higher average number of both major and minor events in the three months preceding relapse compared to the average of other three-month periods in the one-year follow-up (1.9 vs 1.1) but this was not significant (*P* < 0.06). Stressful life events were related to housing, finance, health, employment/education and family/interpersonal areas. Those who relapsed had higher numbers of major events relating to employment/education (M = 1.8) and higher numbers of minor events relating to family/interpersonal sphere (M = 1.6) than those who did not relapse. Only minor events relating to the family/interpersonal sphere were significant (*P* < 0.06).
Megna et al. (2005) USA	Cross-sectional *N* = 31 (M: 54.8%, F: 45.2%)	40.4 (±9.4)	31 Schizophrenia or schizoaffective disorder DSM-IV				Hospitalisation/duration of illness	PLES (Holmes & Rahe, 1968)	Linear regression analysis Annually, 92% less psychosocial stress results in relapse
Serban (1975) USA	Cross-sectional *N* = 641 (acute M: 65, acute F: 60) (chronic M: 329, chronic F: 187)		125 acute schizophrenia and 516 chronic schizophrenia DSM-II		Acute: 60 (48) White, 57 (45.6) Black, 5 (4) Puerto Rican, 2 (1.6) Oriental, 1 (0.8) Other Chronic: 292 (56.6) White, 192 (37.2) Black, 22 (4.3) Puerto Rican, 1 (0.2) Oriental, 9 (1.7) Other	Groups IV and V of the Hollingshead & Redlich Socioeconomic Classification Index	Hospitalisation	SSFIPD	Point-biserial correlation 349 from the original 516 sample of those with chronic schizophrenia were available at the 2-year follow-up. Of the 349, 258 (73.9%) were re-hospitalised. 70 from the original 125 sample of those with acute schizophrenia were available at the 2-year follow-up. Of the 70, 31 (44.3%) were re-hospitalised. Chronic patients scored significantly higher scores on areas relating to interpersonal adjustment (*P* < 0.01) and social adjustment (*P* < 0.05) and these areas were also significantly correlated with re-hospitalisation in chronics (*P* < 0.05) and (*P* < 0.05), respectively. Stress was not correlated with mental status and did not predict re-hospitalisation.
Unal et al. (2019) Turkey	Longitudinal cohort (5-year follow-up) *N* = 147 (M: 89, F: 58)	44.04 (±10.4)	112 schizophrenia, 20 schizoaffective, 15 other psychotic disorders DSM-IV			17 (11.5) high, 44 (30) middle, −86 (58.5) low	8-point criteria, including hospitalisation (Csernansky et al. 2002)	Evaluation of Wide Social Environment based on neighbourhood of patients	Chi-squared, t-test, logarithmic binomial regression At 5-year follow up, 90 (61.2%) had relapsed at least once. Those who relapsed were more likely to be single or divorced (76.7% and 45.6%) and more likely to have moved address at least once (23.3% and 10.5%). Compared to those who did not relapse, those who did were from low-income households.
Vallejos et al. (2017) Argentina	Cross-sectional *N* = 51 (M: 51)	41.27 (±12.61)	51 Schizophrenia DSM-IV			88.2% unemployed	Hospitalisation	ACE (Felitti et al. 1998)	ANOVA, Wilcoxon ranksum test, Mann-Whitney U Four (7.8%) experienced no relapse, 10 (19.6%) were hospitalised once and 37 (72.6%) were hospitalised twice or more. At least one adverse experience during childhood was reported by 48 (94%) patients, and four or more disruptive events were reported by 32 (63%) of the patients. Some of the adverse events included divorce or death (reported by 30 patients, 58.8%), emotional neglect (reported by 28 patients, 54.9%), physical neglect (22 patients, 43.6%). Only 10 patients (19.6%) reported sexual abuse. 94% of the sample reported traumatic childhood events.

*ACE* Adverse Childhood Experiences, *CAS* Composite Abuse Scale – Short, *CCS* Community Care Schedule, *CECA* Childhood Experience of Care and Abuse Questionnaire, *ISSI* Interview Schedule for Social Interaction, *LEDS* Life Event and Difficulty Schedule, *LTE-Q* List of Threatening Experiences Questionnaire, *PCS* Perceived Criticism Scale, *PERI* Psychiatric Epidemiology Research Interview Life Event Schedule, *PLES* Paykel Life Events Schedule, *PRS* Perceived Racism Scale, *SSFIPD* Social Stress and Functionality Inventory for Psychotic Disorders, *SSRQ* Social Readjustment Rating Scale.

### Sample characteristics

The sample characteristics of the 16 included studies are presented in Table 1. These studies included 2248 participants with psychosis. Of these 2248 individuals, 1463 had schizophrenia, 76 had schizoaffective disorder, 24 has psychosis not otherwise specified, 31 had schizophrenia or schizoaffective disorder and 51 were at risk of postpartum psychosis. The participants’ age ranged from 18 to 65 years old, and the majority were male. Furthermore, the measurement of socioeconomic status was only reported in 7 studies.

### Risk of bias assessment scores

The risk of bias assessment scores for all the included studies is shown in Tables 2 and 3. The risk of bias for the 11 cohort studies was also conducted using the NOS. The overall risk of bias score suggests a moderate risk of bias, indicating that these are moderate-quality studies. For the 5 cross-sectional studies, the risk of bias was conducted using the modified NOS. The overall risk of bias score suggests a low risk of bias, indicating these are high-quality studies.

**Table 2:** Risk of bias assessment of cohort studies using the Newcastle-Ottawa scale for assessing studies included in this systematic review.

Study author	Selection				Comparability**	Outcome			Total (9)
	Representativeness of exposed cohort	Selection of non-exposed cohort	Ascertainment of exposure	Demonstration that outcome of interest was not present at start of study*		Assessment of outcome	Was follow-up long enough for outcomes to occur?	Adequacy of follow up	
Hazelgrove et al. (2021)	★	★	–	★	★	★	★	★	★★★★★★★ (7)
Chakraborty et al. (2011)	★	–	–	–*	–	–	★	★	(3)
Docherty et al. (2011)	★	–	★	–*	–	–	★	★	(4)
Chu et al. (1985)	★	–	–	★	–	★	★	★	★★★★★ (5)
Unal et al. (2019)	★	–	★	★	–	★	★	★	★★★★★★ (6)
Cechnickie & Wojciechowska (2008)	★	–	–	★	–	★	★	–	(4)
Hultman et al. (1997)	★	–	–	★	–	★	★	★	★★★★★ (5)
Malla et al. (1990)	★	–	★	★	–	★	★	★	★★★★★★ (6)
Caton & Goldstein (1984)	★	–	★	★	–	★	★	★	★★★★★★ (6)
Krupinski et al. (1971)	–	–	★	–*	–	★	★	–	(3)
Buck et al. (2019)	★	–	★	★	–	★	★	–	★★★★★ (5)

*Study retrospective in nature.

**Comparability assessed as: one star if study controlled for psychotic disorder diagnosis (when healthy controls included); two stars for age/gender.

★: The study has met the criteria.

–: The study has not met the criteria.

**Table 3:** Risk of bias assessment of cross-sectional studies using a modified version of the Newcastle-Ottawa scale for assessing studies included in this systematic review.

Study author	Selection				Comparability**	Outcome		Total (10)
	Representativeness of the sample	Sample size	Non-respondents	Ascertainment of the exposure*		Assessment of outcome***	Statistical test	
Camacho et al. (2012)	★	–	★	★	–	★★	★	★★★★ (6)
Serban (1975)	★	–	★	★★	–	★	★	★★★★ (6)
Vallejos et al. (2017)	★	–	–	★★	–	★	★	★★★★★(5)
Megna et al. (2005)	★	★	–	★★	–	★★	★	★★★★(7)
Lahniers & White (1976)	★	★	–	★★	–	★	★	★★★★(6)

*Ascertainment of the exposure assessed as: one star for non-validated measurement tool, but the tool is available or described; two stars for a validated measurement tool.

**Comparability assessed as: one star if study controlled for psychotic disorder diagnosis (when healthy controls included); two stars for age/gender.

***Assessment of the outcome assessed as: one star for self-report; two stars for independent blind assessment or record linkage.

★: The study has met the criteria.

–: The study has not met the criteria.

### Psychosis relapse

The studies identified by this review employed different definitions of psychosis relapse. Nine studies defined relapse as re-hospitalization^21,23,29,30,46–50^, whereas 3 studies defined relapse as re-hospitalization and symptom exacerbation^5,51,52^. Furthermore, 2 studies defined psychosis relapse as symptom exacerbation^24,25^, and 2 studies defined psychosis relapse using a specific scale (i.e, the 8-point criterion)^22,53^.

### Social stressors

Sixteen studies examined the impact of social stress on psychosis relapse and were eligible for inclusion. Seven studies investigated conflict within social groups (Including racism, criticism, or illness/death)^21,22,24,46,47,50,52^. Three studies investigated general psychosocial stress including lack of social support and social adjustment^29,30,49^. One study examined minor stressful events^25^, while 2 studies investigated a wide range of social stressors (e.g., finances, social environment, and employment/education issues)^25,48^, two studies investigated housing-related stressors (e.g., change of address)^22,23^, and 1 study investigated childhood adverse events and stress sensitivity^5^.

### Social stress and relapse

Out of the 16 studies, 10 investigated whether individuals with psychosis report a high level of psychosocial stress. Whereas one study reported that individuals with psychosis (i.e., FEP, bipolar disorder, schizophrenia, and schizoaffective) have higher levels of perceived and psychosocial stress than healthy controls or other psychiatric conditions^5^.

Out of the 16 studies, 13 investigated the association between psychosocial stress and psychosis relapse in individuals with psychosis. Of these 13 studies, three reported no significant association between psychosocial stress (i.e., life stress and childhood adverse events) and psychosis relapse in individuals with schizophrenia^29,30,50^. In contrast, 10 studies supported the association between psychosocial stress (e.g., childhood maltreatment, address changes, illness/death in family, employment/education changes or redundancies, property damage, racism, interpersonal conflicts, and divorce) and psychosis relapse in individuals with different psychotic disorders (e.g. schizophrenia)^5,21–25,46,48,49,52^.

### Social withdrawal and psychosis relapse

Out of the 16 studies, 6 investigated whether there is an association between social withdrawal and psychosis relapse in individuals with psychosis. These six studies reported that social withdrawal and similar behaviours were evident in individuals with psychosis (e.g., schizophrenia) before their psychosis relapse^30,47,48,51–53^. For example, individuals with chronic schizophrenia were more likely to relapse due to community social adjustment issues^30^ In contrast, individuals with acute schizophrenia were more likely to relapse due to close relationship conflicts^30^. Another study reported reduced social support during hospitalization but not before re-hospitalization (ref. ^54^. Furthermore, Hultman and Wieselgren^52^ reported that socially isolated individuals who were satisfied with their social life were more likely to relapse than socially isolated individuals keen to increase their social contact.

### Interpersonal sensitivity and psychosis relapse

None of the eligible studies assessed interpersonal sensitivity nor its association with psychosis relapse.

## Discussion

The current systematic review examined the association between psychosocial stress and risk of psychosis relapse in individuals with a psychotic disorder. Most studies reported an association between psychosocial stress and psychosis relapse, as well as social withdrawal and psychosis relapse. However, there was no evidence to establish whether individuals with psychosis have a higher level of interpersonal sensitivity and whether this is associated with psychosis relapse. In addition, the reviewed literature showed that individuals with psychosis experience a higher level of psychosocial stress and social withdrawal compared to healthy controls.

### Psychosocial stress and relapse

Thirteen studies provided information regarding the association between psychosocial stress and psychosis relapse in individuals with psychosis. Of these 13 studies, 10 support an association between psychosocial stress (including different types of minor and major social stressors) and psychosis relapse^5,21–25,46,48,49,52^. Two social stressors (i.e., interpersonal conflict and moving home) were found to increase psychosis relapse^22–25^. However, these studies did not document living conditions (e.g., homelessness) preceding psychosis relapse nor the reasons behind address changes, which would have allowed a better understanding of the impact of this form of psychosocial stresses on relapse. Camacho and colleagues reported a contrasting finding indicating that interpersonal conflict has a low impact on hospitalization^21^. A possible explanation for this finding could be the lack of a validated measurement tool to assess social stressors, as individual chart reviews were employed to assess stressors.

Although most studies support the association between psychosocial stress and psychosis relapse, three studies reported contradictory findings^29,30,50^. This could be explained in part by at least two factors: the relatively small sample size (*N* = 51), and the retrospective nature of these studies, which increases the chance of recall bias^55^. In addition, some of these studies used subjective measurement tools (e.g., clinical notes) to assess psychosis relapse. For example, Vallejos et al.^50^ used a self-report questionnaire to assess psychosis relapse. This type of instrument increases the chances of recall bias, as well as under or over-reporting. Finally, Lahniers & White^29^ recruited individuals with acute schizophrenia and did not include individuals in remission or chronic stages of psychosis, which further limits the generalizability of the findings.

### Social withdrawal and relapse

All the included six studies suggest that social withdrawal is associated with psychosis relapse^30,47,48,51–53^. Hultman et al.^52^ reported that socially isolated individuals who were satisfied with their social life were more likely to relapse than socially isolated individuals keen to increase their social contact. A possible explanation is that isolated individuals dissatisfied with their social life may be more vulnerable to relapse because they have lower stress thresholds couples with increased stress-sensitivity, which is consistent with the vulnerability-stress model^17^.

### Interpersonal sensitivity and relapse

The current review search was conducted systematically in three of the most comprehensive databases. Nevertheless, we did not find studies investigating interpersonal sensitivity or its association with psychosis relapse that met our inclusion criteria. Numerous studies examined interpersonal sensitivity among CHR individuals; however, there is a dearth of studies examining interpersonal sensitivity among individuals with a psychotic disorder^34,35^.

### Strengths and limitations

The current review has some limitations. Firstly, due to the findings’ heterogeneity, it was not possible to conduct a meta-analysis alongside the present review. Once more evidence become available, future studies would benefit from employing a meta-analytic approach to quantify the magnitude of associations between variables. Secondly, as variable definitions of relapse were presented across studies, it was difficult to compare findings. Nevertheless, this review helps to address previous reviews’ limitations, as they only used one definition to define relapse, limiting the generalizability of their findings. Thirdly, many included studies were cross-sectional. While this allows the association between these review variables to be examined, longitudinal studies examining the prospective impact of social stress on psychotic relapse would be better suited to clarify this association.

Despite these limitations, this review has a number of strengths. Firstly, this review attempted to address limitations of previous reviews by including studies with a wide range of different stressors and relapse definitions. Secondly, this review included 16 modest-quality studies that have been published recently (from inception until 28th February 2022) in 8 countries, with relatively large sample sizes (*N* = 21–641). The relatively large samples and the geographical distribution contribute to increase the generalizability of findings.

### Clinical implications and future directions

The current review’s findings provide a rationale for mental health professionals to apply preventative interventions to enhance psychosocial stress management skills in individuals with psychosis, and potentially reducing the likelihood of psychosis relapse. Surprisingly, however, none of the established psychological interventions for psychosis, including those which are recommended by NICE, specifically target psychosocial stress. Interestingly, this review included a study that employed a smartphone app to monitor social stress in real time^53^. The use of smartphone apps to track psychosocial stressors in real time might be a possible way of assisting clinicians in the prediction of psychosis relapse^56^. Furthermore, this review highlighted the substantial impact of address change on psychosis relapse, which should be further investigated, collecting detailed information about living conditions before, during, and after the onset of symptoms.

## Conclusions

This systematic review supports the idea of an association between psychosocial stress and risk of psychosis relapse in individuals with a psychotic disorder. Individuals with psychosis tend to experience a high level of psychosocial stress and social withdrawal, and they both appear to increase the risk of psychosis relapse. The role of interpersonal sensitivity in psychosis relapse remains to be explored. Due to the high heterogeneity within the existing literature, only a narrative synthesis of the findings was conducted. Future studies would benefit from employing a meta-analytic approach.

## Methods

### Protocol and registration

This review followed the preferred reporting items for systematic reviews and meta-analyses (PRISMA) guidelines^57^. The review protocol was registered in the prospective international register of systematic reviews (PROSPERO: CRD42021264478), and it documented and specified the methods and exclusion/inclusion criteria in advance.

### Search strategy and selection criteria

We systematically searched Ovid (PsycINFO, EMBASE, MEDLINE) literature databases from inception until 28^th^ February 2022. We employed the following search strings: (psychosis OR psychotic OR schizophreni*) AND (interpersonal sensitiv* OR interpersonal awareness OR relational sensitiv* OR social withdrawal OR social avoidance OR social network OR social stress* OR social advers* OR psychosocial stress*) AND (relapse OR hospitalisation OR readmission OR hospital admission). Hand-searching was also conducted while reviewing the articles’ full text to find new references.

We applied no restrictions regarding ethnicity, gender, socioeconomic status, publication’ date, language, and psychiatric treatment settings (inpatients or outpatients). We applied the following inclusion criteria: (1) randomized controlled trials (RCTs), cohort, case-control, and cross-sectional studies; (2) that assess (Psycho)social stress and psychosis relapse, with psychosis relapse defined as return/exacerbation of psychosis symptoms or re-hospitalization (3); with participants who suffer from a psychosis disorder as indicated by the DSM or ICD, who are between 14 and 65 years old. We excluded (1) qualitative studies, systematic reviews, meta-analyses, and case reports; (2) studies that included participants diagnosed with psychosis due to organic causes or who had a comorbid learning disability; and (3) papers that were unrelated to our variables of interest, namely interpersonal sensitivity, social withdrawal, and psychosocial stress with relapse of psychosis.

### Data extraction process

The study screening was completed independently by one reviewer (KM) and was subsequently cross-checked by a second reviewer (PR). Disagreements were settled by consensus. Furthermore, one reviewer (KM) independently extracted the following data from the included studies: country of origin, author, publication date, study design, sample size, data analysis, key results, participant information, and antipsychotic adherence status. In addition, information about the independent variables (i.e., psychosocial stress, social withdrawal, interpersonal sensitivity) and the dependent variables (i.e., relapse) and how they were measured was extracted.

### Risk of bias assessment

The risk of bias and certainty assessment was conducted by co-authors (KM). The risk of bias for cohort studies was conducted using the modified version of the Newcastle-Ottawa Quality Assessment Scale (NOS) to ascertain the reliability and validity of these studies’ results^58^. The risk of bias for the cross-sectional studies was also conducted using an adapted version of the NOS^59^. In contrast, the risk of bias for RCTs was conducted using a critical appraisal tool^60^.

## Data Availability

Data sharing is not applicable to this article as no new data were collected or analysed as part of this study.
